# Supplementary material for: Identification of SARS-CoV-2 inhibitors targeting Mpro and PLpro using in-cell-protease assay
Source: Commun Biol. 2022 Feb 25;5:169. doi: 10.1038/s42003-022-03090-9 (PMC8881501; doi:10.1038/s42003-022-03090-9)
Supplement: Supplementary file 2 — Supplementary information [file 42003_2022_3090_MOESM2_ESM.pdf]

Supplementary information

Supplementary Table S1: Compounds used to screen inhibitors of Mpro and PLpro by ICP assay

#	Inhibitor	Target
1	2-cyano-Pyrimidine	Cysteine Protease
2	Abacavir sulfate	Reverse Transcriptase
3	Abametapir	metalloproteinase
4	Alogliptin（SYK-322）benzoate	DPP-4
5	Aloxistatin	Cysteine Protease
6	Amprenavir	HIV Protease
7	Arbutin	Tyrosinase
8	Asunaprevir	HCV Protease
9	Atazanavir Sulfate	HIV Protease
10	AZ 960	JAK
11	Biphenyl-4-sulfonyl chloride	HDAC
12	BMS-707035	Integrase
13	Boceprevir	HCV Protease
14	CA-074 methyl ester (CA-074 Me)	Cysteine Protease
15	Calpeptin	Cysteine Protease
16	Carfilzomib (PR-171)	Proteasome
17	Cathepsin Inhibitor 1	Cysteine Protease
18	Cetilistat	Lipase
19	Daclatasvir (BMS-790052)	HCV Protease
20	Daclatasvir Dihydrochloride	HCV Protease
21	Danoprevir (ITMN-191)	HCV Protease
22	Darunavir	HIV Protease
23	Darunavir Ethanolate	HIV Protease
24	Dasabuvir(ABT-333)	HCV Protease
25	Elbasvir	HCV Protease
26	Entecavir Hydrate	Reverse Transcriptase
27	Favipiravir (T-705)	DNA/RNA Synthesis
28	Fosamprenavir calcium salt	HIV Protease
29	Genkwanin	African swine fever virus
30	Glecaprevir	HCV Protease
31	Grazoprevir	HCV Protease
32	Isochlorogenic acid A	HBV
33	Isochlorogenic acid C	HBV
34	Isoxanthohumol	rhinovirus, cytomegalovirus
35	JNJ0966	Matrix metallopeptidase 9
36	Ledipasvir (GS5885)	HCV Protease
37	Leupeptin Hemisulfate	Cysteine Protease, Serine Protease
38	Linagliptin	DPP-4
39	Lithospermic acid	HIV Nucleocapsid

40	Lomibuvir (VX-222, VCH-222)	HCV Protease
41	Lopinavir	HIV Protease
42	Loxistatin Acid (E-64C)	Cysteine Protease
43	Lycorine hydrochloride	HCV Protease
44	MG-101 (ALLN)	Cysteine Protease
45	Nelfinavir Mesylate	HIV Protease
46	Nobiletin	MMP
47	Ombitasvir (ABT-267)	HCV Protease
48	Paritaprevir (ABT-450)	HCV Protease
49	PD 151746	Cysteine Protease
50	Pepstatin A	HIV Protease
51	PSI-6206 (RO-2433, GS-331007)	HCV Protease
52	Rilpivirine	Reverse Transcriptase
53	Ritonavir	HIV Protease
54	Saxagliptin	DPP-4
55	Schisandrin C	ACAT
56	Simeprevir	HCV Protease
57	Sitagliptin	DPP-4
58	Telaprevir (VX-950)	HCV Protease
59	Tizoxanide	HCV Protease
60	Tofacitinib (CP-690550) Citrate	JAK
61	Trelagliptin succinate	DPP-4
62	Velpatasvir	HCV Protease
63	Vildagliptin (LAF-237)	DPP-4
64	Z-FA-FMK	Cysteine Protease

Supplementary Table S2.
Primers used in this study

1	Mpro_C145A_F	TTAATGGTTCAGCTGGTAGTGTTGGTTTTAAC
2	Mpro_C145A_R	ACCAGCTGAACCATTAAGGAATGAACCC
3	PLpro_C1651A_F	AGATAACAACGCCTATCTTGCCACTGCATTG
4	PLpro_C1651A_R	AAGATAGGCGTTGTTATCTGCCCATTTAATAG
5	qRT_F	GTGAAATGGTCATGTGTGGCGG
6	qRT_R	CAAATGTTAAAAACACTATTAGCATA

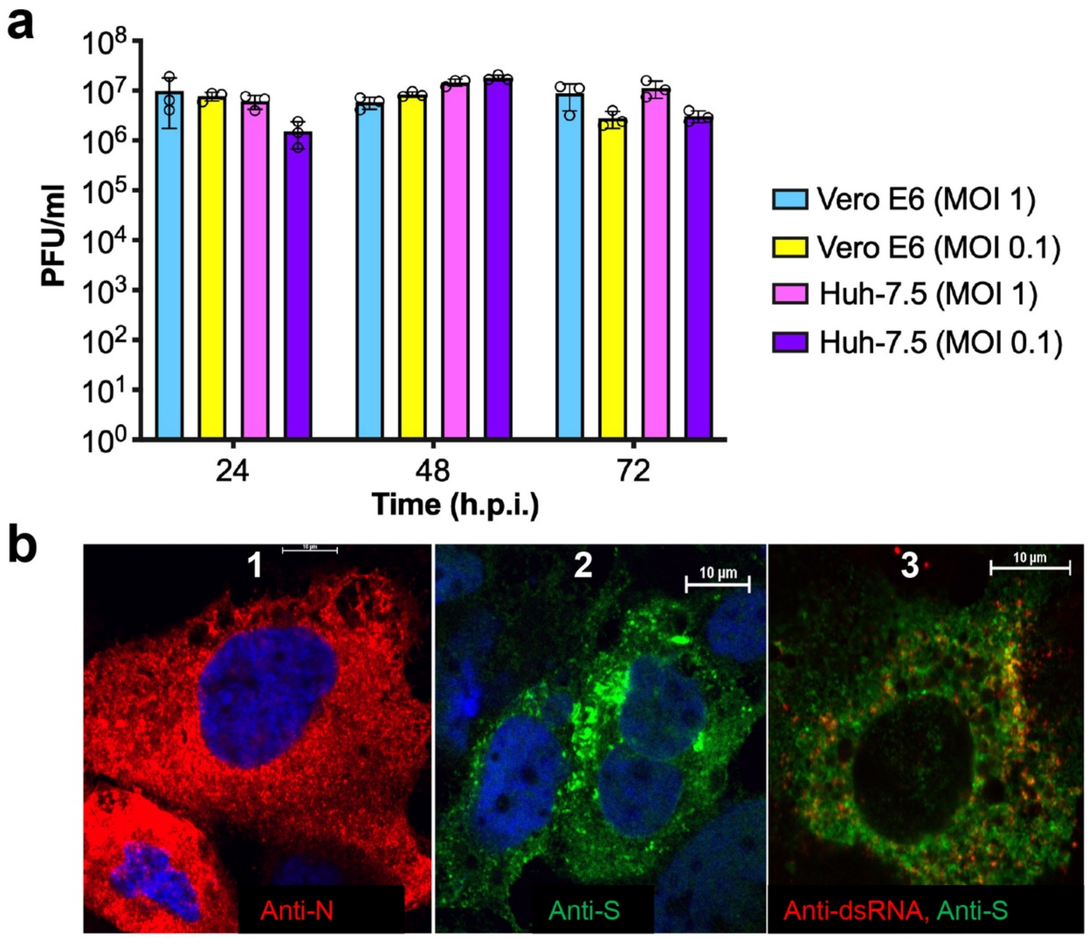

Figure S1. (a) Growth kinetics of SARS-CoV-2. Vero-E6 or Huh 7.5 cells were infected with SARS-CoV-2 with and MOI of 1 or 0.1. Supernatants were collected at indicated hours post infection (h.p.i.) and virus titers were determined by plaque assays using Vero E6 cells. (b) Sub-cellular localization SARS-CoV-2 proteins and replicating RNA in Huh-7.5 cells. Huh-7.5 cells infected with SARS-CoV-2 were fixed at 24 h.p.i. and stained with anti-N (1; red), anti-S (2, 3; green), or anti-dsRNA antibody (English and Scientific Consulting, Szirak, Hungary) (3; red).

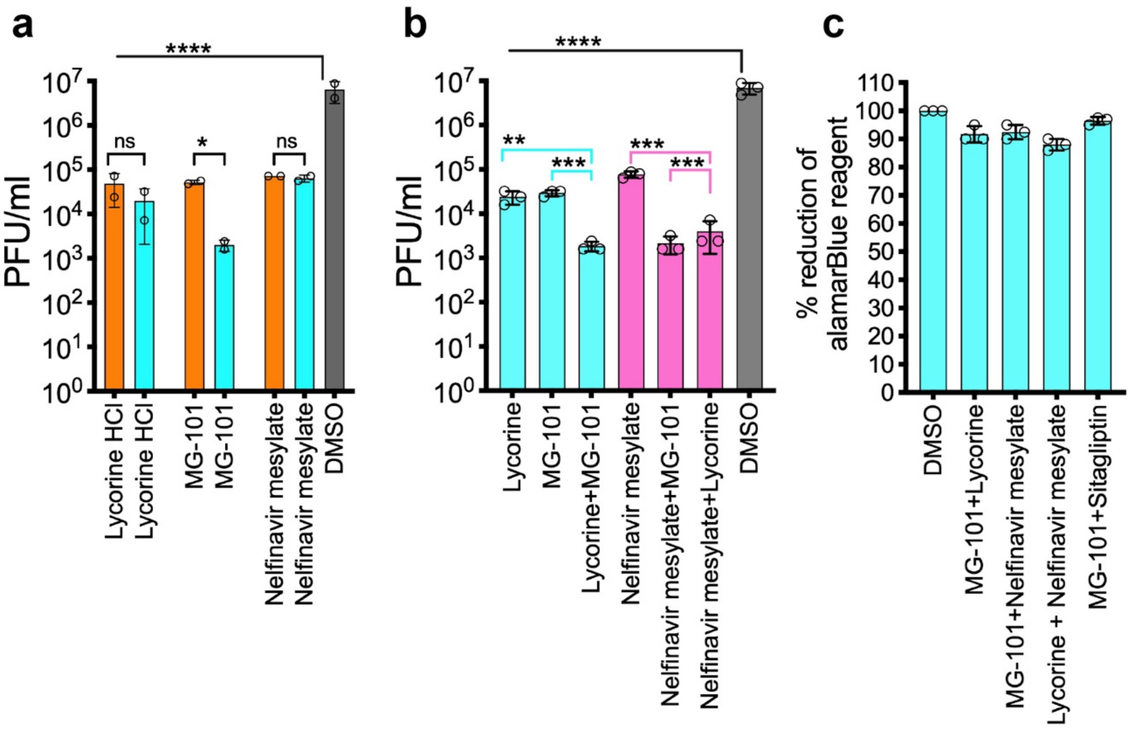

Figure S2. (a) Effect of pre-treatment of compounds on SARS-CoV-2 infection of Huh-7.5 cells. In pretreated cells, Huh-7.5 cells were treated with compounds for 12 h and infected with virus for 24 h in the presence of compounds. In no pre-treatment, cells were infected with virus for 24 h in the presence of compounds. (b) Reduction in virus titers in Huh-7.5 cells treated with various combinations of Mpro inhibitors at 1 μM final concentration. Reduction in virus titer at 24 h.p.i. was determined by plaque assays. Data shown as mean ± SEM. p values were considered significant when $p<0.05$ (*), $p<0.01$ (**), $p<0.001$(***) and $p<0.0001$(****). (c) Cytotoxicity of inhibitor combinations on Huh 7.5 cells determined by alamarBlue reduction assay.

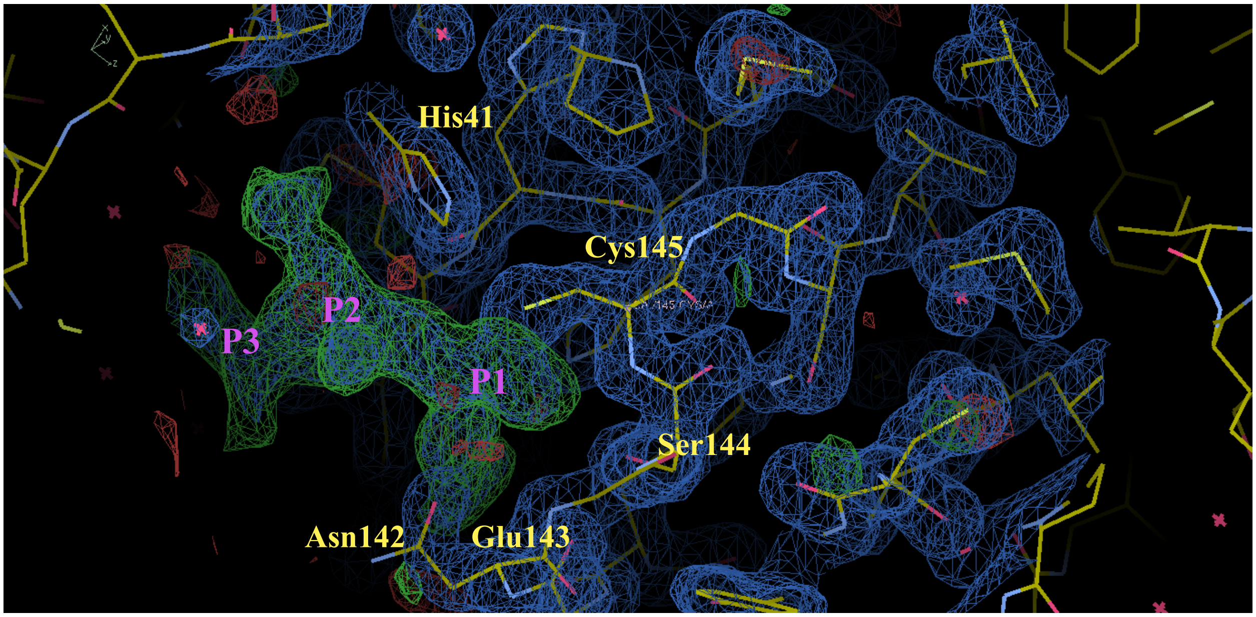

Figure S3. Omit map (green mesh) of MG-101 is shown together with electron density map (blue mesh) of the Mpro and MG-101 complex. Active site cysteine is covalently linked to the P1 position of the peptidomimetic inhibitor MG-101. Catalytic Cys145 and His41 along with the residues comprising the substrate binding site as well as P1-P3 positions of MG-101 are indicated.
