# Supplementary material for: Identification of SARS-CoV-2 inhibitors targeting Mpro and PLpro using in-cell-protease assay
Source: Commun Biol. 2022 Feb 25;5:169. doi: 10.1038/s42003-022-03090-9 (PMC8881501; doi:10.1038/s42003-022-03090-9)
Supplement: Supplementary file 3 — Description of Additional Supplementary Files [file 42003_2022_3090_MOESM3_ESM.pdf]

Description of Additional Supplementary Files

File name: Supplementary Data 1
Description: Source data for the graphs and charts presented in the main figures.
