# Supplementary material for: Identification of SARS-CoV-2 inhibitors targeting Mpro and PLpro using in-cell-protease assay
Source: Commun Biol. 2022 Feb 25;5:169. doi: 10.1038/s42003-022-03090-9 (PMC8881501; doi:10.1038/s42003-022-03090-9)
Supplement: Supplementary file 5 — Reporting Summary [file 42003_2022_3090_MOESM5_ESM.pdf]

nature portfolio

Corresponding author(s): Joyce Jose

Last updated by author(s): Jan 4, 2022

Reporting Summary

Nature Portfolio wishes to improve the reproducibility of the work that we publish. This form provides structure for consistency and transparency in reporting. For further information on Nature Portfolio policies, see our Editorial Policies and the Editorial Policy Checklist.

Statistics

For all statistical analyses, confirm that the following items are present in the figure legend, table legend, main text, or Methods section.

n/a	Confirmed	
☐	☒	The exact sample size (*n*) for each experimental group/condition, given as a discrete number and unit of measurement
☒	☐	A statement on whether measurements were taken from distinct samples or whether the same sample was measured repeatedly
☒	☐	The statistical test(s) used AND whether they are one- or two-sided<br>*Only common tests should be described solely by name; describe more complex techniques in the Methods section.*
☒	☐	A description of all covariates tested
☒	☐	A description of any assumptions or corrections, such as tests of normality and adjustment for multiple comparisons
☐	☒	A full description of the statistical parameters including central tendency (e.g. means) or other basic estimates (e.g. regression coefficient) AND variation (e.g. standard deviation) or associated estimates of uncertainty (e.g. confidence intervals)
☐	☒	For null hypothesis testing, the test statistic (e.g. *F*, *t*, *r*) with confidence intervals, effect sizes, degrees of freedom and *P* value noted<br>*Give P values as exact values whenever suitable.*
☒	☐	For Bayesian analysis, information on the choice of priors and Markov chain Monte Carlo settings
☒	☐	For hierarchical and complex designs, identification of the appropriate level for tests and full reporting of outcomes
☒	☐	Estimates of effect sizes (e.g. Cohen's *d*, Pearson's *r*), indicating how they were calculated

Our web collection on statistics for biologists contains articles on many of the points above.

Software and code

Policy information about availability of computer code

Data collection	crystallography, HKL2000 (v718), molecular operating environment 2020, Schrodinger 2021- 1
Data analysis	Phenix (1.18.2), Coot (0.9.3)

For manuscripts utilizing custom algorithms or software that are central to the research but not yet described in published literature, software must be made available to editors and reviewers. We strongly encourage code deposition in a community repository (e.g. GitHub). See the Nature Portfolio guidelines for submitting code & software for further information.

Data

Policy information about availability of data

All manuscripts must include a data availability statement. This statement should provide the following information, where applicable:

- Accession codes, unique identifiers, or web links for publicly available datasets
- A description of any restrictions on data availability
- For clinical datasets or third party data, please ensure that the statement adheres to our policy

Structural coordinates of structure factors of X-ray crystal structures of Mpro were deposited Protein Data Bank (PDB). Computational data are available on request

Field-specific reporting

Please select the one below that is the best fit for your research. If you are not sure, read the appropriate sections before making your selection.

☒ Life sciences ☐ Behavioural & social sciences ☐ Ecological, evolutionary & environmental sciences

For a reference copy of the document with all sections, see nature.com/documents/nr-reporting-summary-flat.pdf

Life sciences study design

All studies must disclose on these points even when the disclosure is negative.

Sample size	Sample size was determined for obtaining enough number of diffractions to obtain high quality electron density map.
Data exclusions	During X-ray data processing by HKL2000, diffractions in outliers were excluded as a normal procedure.
Replication	Plaque phenotypes and titers of SARS-CoV-2 were determined with three technical replicates and three biological replicates. Data shown are from three technical replicates of one biological experiment. All attempts at replication were successful.
Randomization	N/A
Blinding	N/A

Reporting for specific materials, systems and methods

We require information from authors about some types of materials, experimental systems and methods used in many studies. Here, indicate whether each material, system or method listed is relevant to your study. If you are not sure if a list item applies to your research, read the appropriate section before selecting a response.

Materials & experimental systems

n/a	Involved in the study	
☐	☒	Antibodies
☐	☒	Eukaryotic cell lines
☒	☐	Palaeontology and archaeology
☒	☐	Animals and other organisms
☒	☐	Human research participants
☒	☐	Clinical data
☒	☐	Dual use research of concern

Methods

n/a	Involved in the study	
☒	☐	ChIP-seq
☒	☐	Flow cytometry
☒	☐	MRI-based neuroimaging

Antibodies

Antibodies used	SARS-CoV-2 specific rabbit polyclonal anti-N antibody (Genetex 135384), anti-dsRNA antibody (English and Scientific Consulting, Szirak, Hungary)
Validation	Specificity of anti-N antibody has been validated by ELISA, IFA and western blot by the manufacturer. Anti ds-RNA antibody has been used for ELISA, dsRNA-immunoblotting, and immunohistochemistry as shown by the manufacturer.

Eukaryotic cell lines

Policy information about cell lines

Cell line source(s)	HEK293T cells from ATCC (ATCC CRL-3216), Vero E6 (ATCC CRL-1586), Huh 7.5 (Dr. Charles. M Rice)
Authentication	HEK293T and Vero E6 Cells were purchased and authenticated from ATCC. Huh 7.5 cells were authenticated by Rice lab. No further validation tests were performed.
Mycoplasma contamination	We tested for mycoplasma and did not detect any mycoplasma contamination.
Commonly misidentified lines (See ICLAC register)	None listed in the register of misidentified cell lines.
